# PARAPARESIS FOLLOWING PERIPHERAL AND CENTRAL NERVOUS SYSTEM LESIONS AFTER A LIGHTNING STRIKE – A CASE REPORT

**DOI:** 10.2340/jrm-cc.v8.42545

**Published:** 2025-04-01

**Authors:** Zoé Moyaux, Maria Ciravegna Fonseca De Melo Bandeira, Idil Gunes Tatar, Gautier Randour, Mathilde Massardier, Vincent Van Pesch, Thierry Lejeune

**Affiliations:** 1 Université catholique de Louvain, Secteur des Sciences de la Santé, Institut de Recherche Expérimentale et Clinique, NeuroMusculoSkeletalLab (NMSK), Brussels, Belgium; 2 Cliniques universitaires Saint-Luc, Service de médecine physique et réadaptation, Brussels, Belgium; 3 Cliniques universitaires Saint-Luc, Service de neurologie, Brussels, Belgium; 4 Cliniques universitaires Saint-Luc, Service de radiologie, Brussels, Belgium

**Keywords:** case report, lightning injuries, peripheral nervous system diseases, spinal cord disease

## Abstract

**Objective:**

To describe a case of paraparesis caused by both peripheral neuropathy and spinal cord injury following a lightning strike, highlighting imaging findings and neurophysiological results to improve understanding and management.

**Design:**

A case report.

**Patient:**

A 29-year-old male without significant medical or surgical history, struck by lightning.

**Methods:**

Neurological evaluation, imaging of the spinal cord, electromyography studies, and somatosensory evoked potential assessments were performed. Imaging findings and peripheral nerve evaluations were compared to the existing literature on lightning-related injuries.

**Results:**

Spinal cord imaging showed hyperintensities with a quadrifocal white matter involvement. Neurophysiological study revealed peripheral motor impairment.

**Conclusion:**

This case documents paraparesis resulting from both central and peripheral nervous system damage following a lightning strike. Unique spinal cord imaging results and neurophysiological studies contribute to the understanding of nerve damage mechanisms. Given the increasing frequency of lightning strikes, these findings may help refining clinical management and patient education strategies.

## LAY ABSTRACT

Lightning strikes, often seen as rare events, affect about 240,000 people worldwide each year, with many surviving. However, they can lead to serious injuries, especially to the nervous system. This article discusses the case of a 29-year-old gardener who was struck by lightning, resulting in weakness of the lower limbs. The patient experienced damage to both components of the nervous systems, presenting a rare combination of injuries. The results from imaging and neurological assessments provide valuable insights into the mechanisms of injury, which can inform treatment strategies and improve patient care. By enhancing our understanding of these complex injuries, we could support better individuals affected by lightning strikes.

Although lightning strikes are often perceived as rare and fatal events, about 240,000 people are struck worldwide annually with a 90% survival rate (1). A wide range of injuries may occur, with the nervous system being particularly susceptible. Neurological disorders associated with such injuries exhibit significant variability in anatomical localization, severity, onset and temporal duration (2–4). Global climate change would lead to more disorders due to increasing frequency in lightning strikes (5). This contrasts with the existing literature, which documents older cases that did not have the advantage of modern diagnostic imaging and neurophysiological techniques. Here, we report a case of paraparesis in a young man following a lightning strike, with both peripheral neuropathy and spinal cord injury (SCI).

## MEDICAL HISTORY

A 29-year-old man with no significant medical or surgical history was struck by lightning during a thunderstorm. He was working as a gardener and took shelter under a tree. He was wearing hearing protection, which was shattered on impact. Immediately, the subject suffered a cardiac arrest. His colleague, who was nearby, promptly contacted emergency services and initiated cardiopulmonary resuscitation (CPR). Upon the arrival of the emergency services, he was found in asystole with a Glasgow Coma Scale score of 3/15. Sinus rhythm was restored after 3 CPR cycles and the subject was sedated and intubated. The no-flow time was estimated to be less than 2 min and the low-flow duration approximately 15 min.

**Fig. 1:**
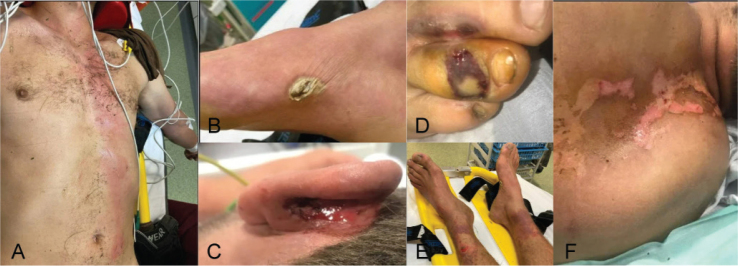
Picture of the cutaneous lesion (A. torso, B. right foot, C. left ear, D. toes right foot, E. ankles, F. back).

At the emergency department, physical examination revealed burns on both feet, ankles, pubic area, torso, back, and left ear (Fig. 1). Blood analysis revealed an elevated creatine kinase (CK) level of 3700 UI/L (range 100–200 UI/L). Cerebral and cervical computed tomography (CT) scans showed no evidence of bleeding or structural injuries. The subject was admitted to the Intensive Care Unit where he remained intubated and sedated for 3 days. A tympanic membrane perforation was detected. Upon awakening on day 3, the subject exhibited paraparesis.

On day 8, the subject was transferred to the neurology ward. The American Spinal Injury Association (ASIA) Impairment Scale (AIS) was assessed AIS C with a motor level L2, a sensory level T11, resulting in a neurological level of injury of T11. He reported bilateral lower limb dysesthesia, hypoesthesia, and hypopallesthesia. The subject presented at-level T11-T12 neuropathic pain requiring pregabalin initiation. Deep tendon reflexes in the lower limbs were absent, and the Babinski sign was absent. At that time, the subject underwent mobilization physiotherapy and demonstrated the ability to maintain a seated position.

**Fig. 2:**
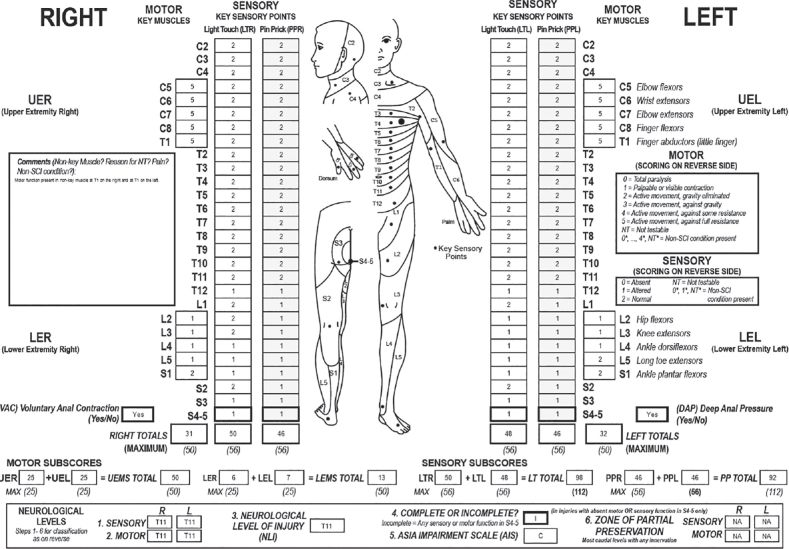
International Standards for Neurological Classification of Spinal Cord Injury (ISNCSCI) examination on the admission in the rehabilitation department.

Upon the admission to the rehabilitation department on day 19, neurological findings remained consistent with a sensory level T11, motor level L2 and neurological level of injury of T11. The ASIA classification was AIS C. The total motor score was 63/100 with a right leg motor score of 6/25 and a left leg motor score of 7/25 (Fig. 2). Despite an initial episode of bladder retention necessitating catheterization, the subject subsequently regained normal voluntary bladder voiding. However, he reported persistent urinary symptoms, including false urinary urgency and abdominal discomfort, which prompted a urodynamic evaluation. This assessment revealed a detrusor hypocontractility, with no evidence of bladder instability and a preserved bladder capacity. Persistent T11–T12 neuropathic pain required an increased dosage of pregabalin. The Spinal Cord Independence Measure (SCIM) score was 78 out of 100, with a particularly low mobility and movement subscore of 18 out of 40. The rehabilitation program included 90 min of physiotherapy and 30 min of occupational therapy each day, 6 day a week. Physiotherapy was focused on muscle strengthening, maintaining the range of motion and learning to handle the wheelchair. During occupational therapy sessions, the patient progressed to a standing position in a standing frame and worked on daily living activities. The subject underwent psychological counselling and scored within the normal range on the Hospital Anxiety and Depression Scale (HADS). Cognitive assessment revealed no deficit, with a Montreal Cognitive Assessment (MoCA) scale of 30/30.

Three months after the event, neurological impairments remained stable, with a neurological level of injury remaining at T11, AIS C, right leg motor score 6/25 and left leg motor score 7/25. The neuropathic pain diminished and the patient reported a reduction in episode of false urinary urgency. At that time, the subject went to physiotherapy, which aimed at ambulation, utilizing a weight support system and a walking aid. For daily activities, he employed a manual wheelchair.

Five months post-injury, the neurological level of injury was determined to be T11 with partial recovery of sensation noted: the right sensitivity level improved to L3, while the left remained at T11. The AIS classification remained C, but a slight motor recovery was also observed with a right leg motor score of 9/25 and a left leg motor score of 7/25. The subject demonstrated the ability to walk within his room and during physiotherapy sessions using a walking aid, while continuing to rely on a manual wheelchair for longer distances. Functional independence improved significantly, as reflected by a SCIM score of 91 out of 100. He was then able to return home at weekends with the help of his relatives.

**Fig. 3:**
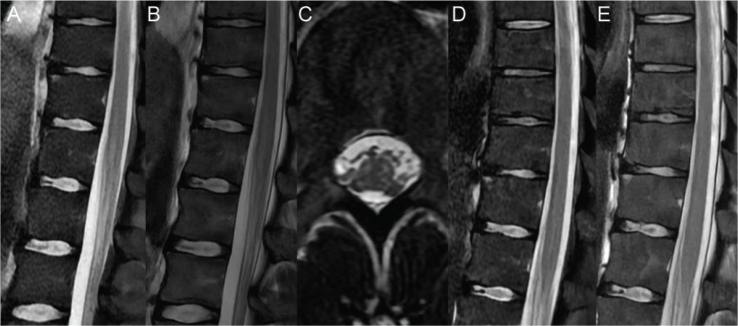
MRI imaging (A. First sagittal Short Tau Inversion Recovery (STIR), B. First magnetic resonance imaging (MRI) sagittal T2, C. First MRI axial T2, D. Second MRI sagittal STIR, E. second MRI sagittal T2).

Spinal cord magnetic resonance imaging (MRI), conducted 4 days post-strike revealed a linear hyperintensity on sagittal T2-weighted and Short Tau Inversion Recovery (STIR) sequences in the conus medullaris, extending approximately 5 cm from T11/T12 to L2 (Fig. 3A/B). Axial T2 weighted images showed focal hyperintensities in a quadrifocal topography of the peripheric white matter in the conus medullaris (Fig. 3C). A follow-up spinal cord MRI, conducted 15 days post-strike, demonstrated a complete resolution of previously observed signal abnormalities on both sagittal T2-weighted and STIR images, with no further anomalies identified (Fig. 3D/E). Additionally, a cerebral MRI conducted 14 days after the strike showed no abnormality. One-month post-injury, a total body muscle MRI was performed, which indicated the absence of muscular necrosis, and no signs of fatty infiltration. Subtle edema was observed in the muscles of the legs and feet muscles.

Lower limb somatosensory evoked potentials (SSEP) performed at 16 days and 11 weeks post-strike, revealed reduced sensory conduction velocities in both legs. Spinal activations were not identified. The primary cortical activities (P37 component) were present but delayed. Motor evoked potentials (MEP) conducted 16 days post-injury revealed delayed responses in the right anterior tibial muscle after cortical and radicular stimulation, with a prolonged central conduction velocity. Responses were absent initially in the left anterior tibial muscle, upon cortical and radicular stimulation. Responses in the upper limbs were normal. Eleven weeks post injury, lower limb MEPs demonstrated delayed responses bilaterally, particularly on the left, upon cortical stimulation. The right radicular response was delayed and the left was still absent. Right central motor conduction time remained prolonged. Both SSEP and MEP are consistent with SCI, in addition to peripheral nerve involvement in both lower limbs.

Two electromyography (EMG) studies were performed. The first EMG, performed 14 days after the injury (Table I), revealed normal sensory conduction in the sural nerve and normal motor conduction velocity in the left fibular and tibial nerves, though the amplitude of compound muscle action potentials recorded at the foot was reduced. Needle EMG performed on the left lower limb showed no abnormal resting activity, and no voluntary activation was detected. The second EMG, performed 31 days after the injury (Table I), demonstrated a bilateral reduction in the amplitude of sensory nerve action potentials in the sural nerves. Motor conduction velocity of the left fibular and tibial nerves was slightly reduced with low amplitude of compound muscle action potentials of the intrinsic foot muscles. Needle EMG showed widespread fibrillation and positive sharp waves in all examined lower limb muscles and no voluntary activation was detected. At both examinations, motor and sensory nerve conduction and needle EMG studies were normal in the upper limbs. These 2 consecutive EMG demonstrate an acute, axonal, motor and sensory peripheral neuropathy affecting both lower limbs.

**Table I:** Electromyography (EMG) results

Electromyography	First EMG (Day 15)				Second EMG (Day 31)			
Motor Nerve conduction								
Ulnar – ADM	Latency	Duration	Amplitude	Speed	Latency	Duration	Amplitude	Speed
Wrist	2.6	6.6	9.8	61	2.6	7.0	9.3	61
Elbow	6.9	6.8	9.6		6.4	7.4	7.8	
Peroneal – EDB								
Ankle	3.9	5.5	0.5	51	4.2	8.8	0.3	39
Fibula head	11.0	5.9	0.3	42	12.6	10.7	0.3	44
Knee	13.3	6.6	0.3		14.6	9.9	0.3	
Tibial – AHB								
Ankle	4.9	4.0	3.8	42	5.3	5.4	1.4	39
Knee	14.1	5.7	2.1		15.7	6.7	0.8	

Sensory nerve conduction	Onset Latency	Peak Latency	Amplitude	Speed	Onset Latency	Peak Latency	Amplitude	Speed
Median L. – Wrist D2	2.2	3.1	57		2.3	3.1	47	56
Ulnar L. – Wrist – D5					2.1	2.9	34	53
Sural L. – Calf – Ankle	3.3	4.4	7		NR	NR	NR	NR
Sural R. – Calf – Ankle					NR	NR	NR	NR

Needle EMG	Fib – PSW	MUAP morphology	Recruitment	Activation	Fib – PSW	MUAP morphology	Recruitment	Activation
Biceps Brachii	0	0	Nl	Nl	0	0	Nl	Nl
Tibialis Anterior	0	0		None	+++	+++		None
Medial Gastrocnemius	0	0		None	+++	+++		None
Vastus Medialis	0	0		None	+++	+++		None

ADM: Abductor Digiti Minimi; AHB: Abductor Hallucis Brevis; EDB: Extensor Digitorum Brevis; MUAP: Motor Unit Action Potential; PSW: Positive Sharp Wave

## DISCUSSION

Lightning is a widespread natural phenomenon and the second leading cause of weather-related death, with an estimated 24,000 fatalities annually. Most lightning strikes are nonlethal, with approximately 10 survival cases for every reported fatality (1).

Five mechanisms of lightning injury are documented: direct strike, contact voltage, side splash, step voltage and upward streamer (4). Determining the exact mechanism in our case is challenging, but possible mechanisms include contact voltage through the earmuffs, side splash from the tree, or step voltage.

Cardiac, pulmonary, dermatological, ophthalmologic, and otologic injuries are well-documented. The cardiac and auricular injuries observed in our subject are consistent with those reported (4). This was also the case for skin lesions (linear, punctate and thermal), including the characteristic toe burns (4, 6). The punctate lesion on the right foot probably corresponds to an entry/exit point (6). However, the classic Lichtenberg burn was not present (6–8). A wide range of neurological lesions has been described in the literature (2–4). However, there is limited literature on lightning-induced spinal cord injuries and peripheral nerve disease (9, 10). Cherington et al. developed a classification system based on the onset, duration, and severity of symptoms, which remains in use today (2). We focused on the second category of this classification, which includes immediate and prolonged or permanent injuries.

### Spinal cord injury

Four cases of paraplegia have been described in the literature, with neurological injury levels varying between T8 and L2 (3, 9, 11, 12). One case indicated that the level of the burn lesion corresponded to the neurological level of injury, suggesting that spinal cord damage may align with the path of the electrical current (9). Most cases exhibited diminished vibration sense, reduced pinprick sensation, and altered temperature perception (11, 12), possibly linked to an anterior and posterior white matter lesion. MRI findings were available for only 1 subject with flaccid paraplegia and sensory deficits and revealed necrosis of the anterior horn at the L2–L3 level, as evidenced by an anterolateral hypo-intensity on T2-weighted sequences (3). In our case, MRI revealed quadrifocal involvement of the white matter, supporting the presence of a direct nerve lesion caused by electrical conduction, rather than the previously hypothesized vascular ischemia (13). Although a vascular hypothesis was initially considered, given the preceding cardiac arrest and previous literature implicating vascular mechanisms in similar pathophysiology (11), the observed MRI pattern was inconsistent with ischemic infarction. Spinal cord ischemia is expected to involve primarily the anterior horns or posterior columns, depending on the arterial territory involved, with the possibility of surrounding white matter involvement. In our case, the main involvement is in the peripheral white matter, which is confirmed on transverse imaging by the absence of hypersignal in the central gray matter. The owl’s eye sign, vertebral body infarction and predominant gray matter involvement were notably absent. In addition, the enhancement typically expected between 8 and 17 days post-injury was not observed (12).

This is also the first documented case where an MRI abnormal signal returns back to normal. It highlights the importance of conducting prompt MRI evaluations following a lightning strike, especially when neurological symptoms are present, as these can provide critical insight into the nature of the injury. Furthermore, this case adds valuable data to the existing imaging literature regarding spinal cord injuries in subjects struck by lightning.

### Peripherical nerves lesions

Hawkes et al. (13) described extensive peripheral nerve damage in a quadriplegic subject. Bilateral median nerve neuropathy was characterized by absent sensory nerve action potentials and prolonged terminal motor latency. Cortical magnetic stimulation failed to evoke a potential from the abductor digiti minimi (MEP). Needle EMG showed denervation of the intrinsic hand muscles. Morin et al. (3) described the case of a woman with sensory symptoms in upper limbs, with EMG showing low sensory and motor amplitude in the median nerves. The only reported severe, albeit patchy, axonal loss peripheral motor and sensory neuropathy is a man struck by lightning who was resuscitated after 50 min and remained 53 days in the intensive care unit with paraparesis of the lower limbs. Our case represents the first well-documented instance of neurological peripheral nerve lesions in the lower limbs. The absence of upper limb involvement supports the hypothesis that the electrical current predominantly traversed the lower limbs. The symptoms attributed to these lesions are challenging to delineate due to the concurrent SCI. This case may serve as a valuable example of neuropathy within the context of lightning strikes.

### Muscle damage

CK levels are frequently elevated and compartment syndrome has been reported in 2 instances where the subjects underwent fasciotomy to relieve increased compartment pressure. While muscle damage is documented in the literature, the symptoms exhibited by our subject do not align with those typically associated with muscle injury (14).

### Neurological pain

Few publications exist about pain in victims of lightning injuries. In this case, significant neuropathic pain localized to the T11–T12 region prompted an increase in pregabalin dosage, which subsequently alleviated the pain. This observation aligns with the hypothesis that such pain is derived from direct peripheral nerve and/or receptor cell damage.

### Psychological and cognitive status

Cognitive assessments, as well as evaluations for anxiety and depressive disorders, yielded results within the normal range. However, it should be noticed that lightning injuries can cause psychiatric symptoms, such as post-traumatic stress disorder (PTSD), anxiety, and depression, which may contribute to cognitive dysfunction (4).

### Follow-up

To date, no cases in the existing literature have been systematically documented using the AIS or functional assessment tools such as the SCIM. Although the follow-up period in this case was relatively short, it provides valuable preliminary insights into the trajectory of neurological recovery and functional outcomes.

### Conclusion

This case underscores the complex nature of injuries from lightning strikes, particularly their neurological consequences, highlighting both SCI and peripheral nerve disorder. Our findings support direct nerve damage as a contributing mechanism to SCI and provide the first documented case of lesion regression following a lightning strike.
